# A highly sensitive and specific real-time quantitative PCR for *BRAF* V600E/K mutation screening

**DOI:** 10.1038/s41598-020-72809-7

**Published:** 2020-10-09

**Authors:** Jrhau Lung, Ming-Szu Hung, Yu-Ching Lin, Yuan Yuan Jiang, Yu-Hung Fang, Ming-Shian Lu, Ching-Chuan Hsieh, Chia-Siu Wang, Feng-Che Kuan, Chang-Hsien Lu, Ping-Tsung Chen, Chieh-Mo Lin, Yen-Li Chou, Chin-Kuo Lin, Tsung-Ming Yang, Fen Fen Chen, Paul Yann Lin, Meng-Jer Hsieh, Ying Huang Tsai

**Affiliations:** 1 Department of Medical Research and Development, Chang Gung Memorial Hospital, Chiayi Branch, Chiayi, Taiwan; 2 Department of Pulmonary and Critical Care Medicine, Chang Gung Memorial Hospital, Chiayi Branch, Chiayi, Taiwan; 3 Department of Medicine, College of Medicine, Chang Gung University, Taoyuan, Taiwan; 4 Department of Respiratory Care, Chang Gung University of Science and Technology, Chiayi Campus, Chiayi, Taiwan; 5 Division of Thoracic and Cardiovascular Surgery, Department of Surgery, Chang Gung Memorial Hospital, Chiayi Branch, Chiayi, Taiwan; 6 Department of General Surgery, Chang Gung Memorial Hospital, Chiayi Branch, Chiayi, Taiwan; 7 Department of Hematology and Oncology, Chang Gung Memorial Hospital, Chiayi Branch, Chiayi, Taiwan; 8 Department of Pathology, Chang Gung Memorial Hospital, Chiayi Branch, Chiayi, Taiwan; 9 Department of Anatomic Pathology, Dalin Tzu Chi Hospital, Buddhist Tzu Chi Medical Foundation, Chiayi, Taiwan; 10 Department of Respiratory Care, College of Medicine, Chang Gung University, Taoyuan, Taiwan; 11 Department of Pulmonary and Critical Care Medicine, Chang Gung Memorial Hospital, Linkou Branch, Linkou, Taiwan

**Keywords:** Cancer, Medical research

## Abstract

Mutations that lead to constitutive activation of key regulators in cellular processes are one of the most important drivers behind vigorous growth of cancer cells, and are thus prime targets in cancer treatment. *BRAF* V600E mutation transduces strong growth and survival signals for cancer cells, and is widely present in various types of cancers including lung cancer. A combination of BRAF inhibitor (dabrafenib) and MEK inhibitor (trametinib) has recently been approved and significantly improved the survival of patients with advanced NSCLC harboring *BRAF* V600E/K mutation. To improve the detection of *BRAF* V600E/K mutation and investigate the incidence and clinicopathological features of the mutation in lung cancer patients of southern Taiwan, a highly sensitive and specific real-time quantitative PCR (RT-qPCR) method, able to detect single-digit copies of mutant DNA, was established and compared with BRAF V600E-specific immunohistochemistry. Results showed that the *BRAF* V600E mutation was present at low frequency (0.65%, 2/306) in the studied patient group, and the detection sensitivity and specificity of the new RT-qPCR and V600E-specific immunohistochemistry both reached 100% and 97.6%, respectively. Screening the *BRAF* V600E/K mutation with the RT-qPCR and V600E-specific immunohistochemistry simultaneously could help improve detection accuracy.

## Introduction

Somatic mutations caused by various insults during life-time are one of the most important factors driving normal cells out of control and to become cancer cells. Mutations in key regulators of cellular processes tend to be selected and kept within genome during tumorigenesis, if the mutation brings growth or survival advantages. Gain-of-function mutations are frequently clustered at specific regions or domains of encoded proteins and are important targets for cancer targeted therapy^1,2^. *EGFR* and *KRAS* mutations, and fusion genes such as *ALK*, *ROS1*, and *RET*, are the most representative examples in lung cancers. Many treatments specific against these mutant proteins have shown effectiveness in attenuating cancer progression and improving significantly survival of cancer patients. Continued identification and verification of new driver mutations, and development of corresponding target therapy drugs are keys to effective cancer treatment.

BRAF, an important serine/threonine kinase in RAS/RAF/MEK/ERK pathway, transduces signals from various stimuli and triggers cell growth, proliferation, differentiation, and survival. Owing to its pivotal roles in cellular functions, upregulation of BRAF activity by various mechanisms, including upregulation or mutations in upstream regulators or itself, is frequently found in cancers^3,4^. Currently, more than 200 different types of *BRAF* mutations, which occurred predominately in exons 11 and 15, and translations with great difference in incidence and impact on BRAF kinase activity have been identified from various types of cancers^5^. Not all these *BRAF* mutants would result in elevated enzyme activities and some even impaired activity. Through cooperating with other RAF isoforms, elevated signaling could still be transmitted downstream for those activity-impaired mutants^6^. Reported incidences of *BRAF* mutations in lung cancers ranged from 0.9 to 8.9%. These mutations have been identified in wide ranges of lung cancer histological types, including adenocarcinoma, squamous, and large cell, but tend to be prevalent in adenocarcinoma. Significant associations of these mutations were found with female gender and older ages but not with smoking status and disease stage^5,7^. Among these mutations, the activating *BRAF* V600E (*GTG* > *GAG*) occurs most frequently in lung cancers, which accounts for approximately 50% of all *BRAF* mutation cases, and is preferentially present in female and non-smokers^7^. The prognostic implication of these *BRAF* mutations has not been clearly established, but they tend to associate with poor survival in the early-stage but not advanced-stage lung cancer patients^5,8,9^. Administration of BRAF inhibitors, such as vemurafenib and dabrafenib, have shown to improve the response and survival of patients with *BRAF* V600E mutations regardless of their previous treatment. However, these inhibitors bring no significant benefit for patients with most of the other *BRAF* mutations, except those occurring at the 600^th^ amino acid of BRAF, such as the other frequently observed V600K mutation (*GTG* > *AAG*), which are able to constitutively activate BRAF kinase activity both in monomeric or dimeric forms^10,11^. Despite these improvements, rapid progression and development of secondary skin cancers are frequently observed among patients with these monotherapies. The inadequacies of these treatments were improved after co-administration of dabrafenib with a MEK inhibitor (trametinib)^5,12^. Currently, this combination has been approved by FDA and recommended by ESMO, and would become the preferred treatment option for the V600E-mutated lung cancer^12–14^. This combination was also found to be effective for non-V600-mutated lung cancer in cell line model^15^, but the efficacy in patients is still waiting to be confirmed in clinical setting. Moreover, this combination treatment is still contraindicated or not reimbursement in some countries. Nevertheless, recent studies have indicated that vemurafenib monotherapy could be a feasible alternative for patients in these conditions^11^. Emerging data have also shown that the outcome of these *BRAF* mutated patients could be even better if these target treatments are put in the frontline^16^. Despite this advancement, further treatment optimization is still required to improve the success of BRAF target therapy for lung cancers.

To study the incidence and clinicopathological features of *BRAF* V600E/K mutation in lung cancer patients of southern Taiwan, a highly sensitive RT-qPCR assay was established. Although current high-throughput platforms, such as next-generation sequencing, is more suitable for efficient screening of driver mutations in lung cancers before treatment, they are technically more challenging and have not been widely available and validated. RT-qPCR and immunohistochemistry are still commonly used for detection and serve reliably for molecular diagnosis in clinical practice. The present findings showed low incidence of *BRAF* V600E mutation in the studied lung cancer population, and that the newly established RT-qPCR method and BRAF V600E-specific antibody have high concordance in *BRAF* V600E detection. Screening the *BRAF* V600E/K mutation with these two methods simultaneously could help improve detection accuracy.

## Results

### Development and performance of *BRAF* V600E/K mutation-specific RT-qPCR assay

**Figure 1:**
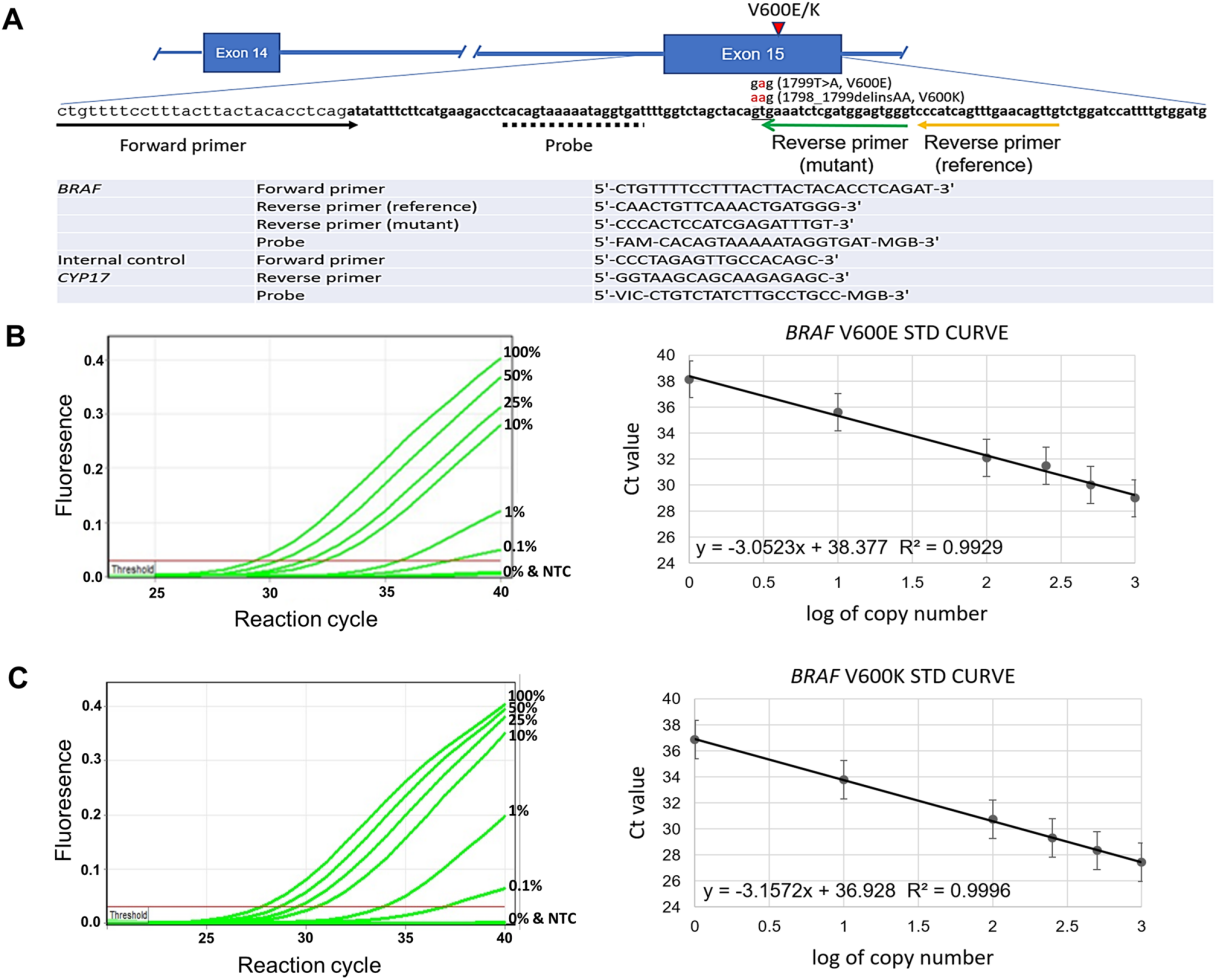
Design and performance of the improved *BRAF* V600E/K mutation-specific RT-qPCR. **(A)** Sequences and relative locations of the primers and probe used in the assay. Amplification plots and standard curves for mutant alleles of *BRAF* V600E **(B)** and V600K **(C)**. The standard curve of threshold cycle values were plotted against the log of copy numbers of mutant alleles and labeled with standard error bars. Each data point represents an average of three detection replicates for each dilution and the linearity is valid over four logs. The amplification plots were snapshots from the Qiagen Rotor-gene Q series software. Other parts of the figure were created using Microsoft Office 2013.

To better recapitulate the real assay condition and evaluate assay formulation, DNA standards containing two to 2000 copies of the *BRAF* V600E allele were prepared using a mixture of genomic DNA from HEK293 (*BRAF* wild type) and HT-29 (*BRAF* V600E heterozygote). The original assay formulation developed by Lang et al.^17^ failed to reach the sensitivity shown in their study, which adopted plasmid-based standard, when performed on the genomic DNA-based standard (data not shown). The template complexity of plasmid-based standard was much lower than that of the cell line genomic DNA-based standard; hence, a qPCR master mix, Qiagen QuantiNova probe master mix, with better DNA polymerase processability and higher yield was used instead for evaluating assay formulation. However, the new master mix adopted abolished the selectivity of the mutant-specific primer and generated strong signals in wild-type controls (Supplemental Fig. S1). For readjustment, various penultimate mismatched nucleotides were introduced to the *BRAF* mutant-reverse primers. The one with G in the penultimate nucleotide showed the best sensitivity and specificity; and was thus chosen for downstream *BRAF* V600E screening (Supplementary Fig. S1). The assay can reliably detect *BRAF* V600E mutant allele in as low as two copies, which corresponds to a detection limit of 0.1% mutant allele in the presence of 99.9% wild-type counterparts. The correlation coefficient of the qPCR standard curve reached 0.99, suggesting the reliability of the newly established assay in a wide range of mutant alleles (Fig. 1). With the developed primer in the assay being able to recognize another frequently found *BRAF* V600K mutation (GTG > AAG), the performance of the assay in detecting the V600K mutation was also evaluated using genomic DNA from V600K-positive melanoma cell line, LM-MEL-42 (V600K heterozygote). Results obtained showed comparable detection sensitivities of the developed RT-qPCR and *BRAF* V600E and V600K, both reaching 0.1% (Fig. 1).

### *BRAF* V600E/K mutation in lung cancer population of southern Taiwan

**Figure 2:**
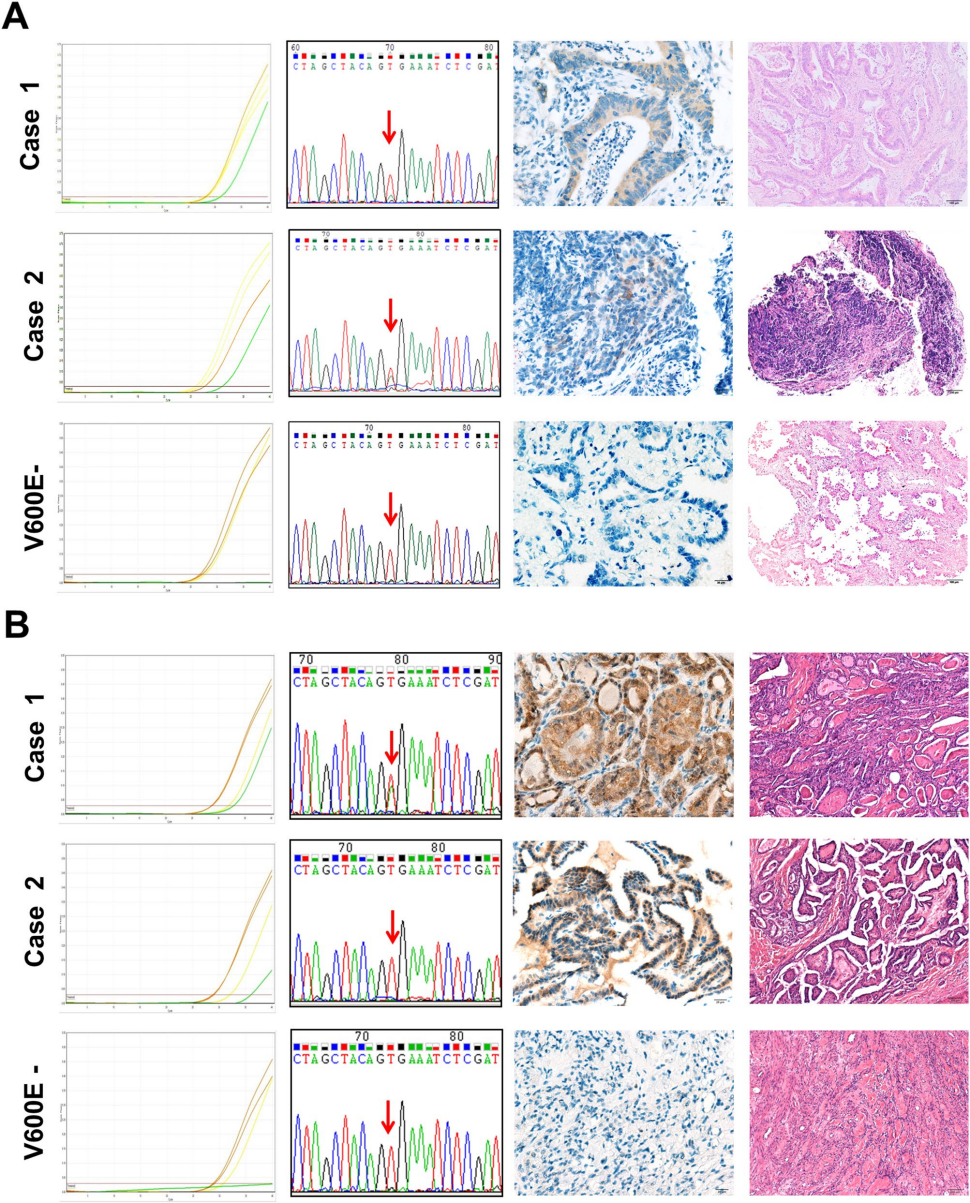
*BRAF* V600E/K mutation-specific RT-qPCR, Sanger sequencing, V600E specific immunohistochemistry and histology staining results for representative cases of lung cancer **(A)** and thyroid cancer **(B)**. Figures in the first column are qPCR amplification plots. Green, orange and yellow curves represent signals corresponding to *BRAF* V600E/K alleles, *BRAF* wild-type allele and *CYP17* genes, respectively. Chromatograms in the second column are Sanger sequencing results and mutation sites are marked with red arrows. Images in the third and fourth columns are immunohistochemistry staining and hematoxylin/eosin staining results, respectively. The amplification plots and chromatograms of Sanger sequencing were snapshots from the Qiagen Rotor-gene Q series software and Chromas lite (v2.21, https://www.technelysium.com.au/), respectively. The immunohistochemistry and hematoxylin/eosin results were captured using Olympus BX51TF microscope and DP80 camera with cellSens software under × 40 and × 10 magnification, respectively.

To study the incidence of *BRAF* V600E/K mutation in a lung cancer population of southern Taiwan, 306 lung cancer samples were analyzed using the established *BRAF* V600E/K mutation-specific RT-qPCR. All samples were performed successfully according to the signals of *CYP17* gene and *BRAF* wild-type allele. Among them, two samples were qPCR-positive and both were found carrying V600E in the subsequent verification by Sanger sequencing (Fig. 2A). Both patients were male with adenocarcinoma of advanced stage (stage IV). One was a 74-year-old non-smoker and the other was a 50-year-old heavy smoker. The incidence of *BRAF* V600E mutation in the studied lung cancer population was 0.65%.

### V600E mutation-specific immunohistochemistry

Immunohistochemistry is commonly used in molecular diagnosis; hence, the performance of a *BRAF* V600E-specific mouse monoclonal antibody (clone VE1) in *BRAF* V600E screening was investigated and compared with the established RT-qPCR assay. In consideration that most of the prospectively collected samples harvested by needle or forceps biopsy were tiny and there was not much left after earlier diagnosis works, only the one *BRAF* qPCR-positive specimen in the prospectively collected sample group was included in the comparison study. The remaining samples evaluated comprised 193 samples requested from the tissue bank and biobank in tissue microarray format as well as the other *BRAF* V600E positive sample. Immunohistochemistry was successfully performed for the 194 samples, and the BRAF V600E-specific antibody stained positively only on the two *BRAF* V600E-mutated samples, one with moderate intensity and the other with weak focal staining (Fig. 2). These results indicated that BRAF V600E specific antibody and the established RT-qPCR assay had a high concordance in *BRAF* V600E detection of lung cancer.

### Detection sensitivity and specificity of RT-qPCR assay and V600E-specific immunohistochemistry

Only two *BRAF* V600E mutation cases were identified from the studied lung cancer population; hence, the performance of the developed RT-qPCR assay and V600E-specific immunohistochemistry could not be well validated. To determine the detection sensitivity and specificity of the two methods in *BRAF* V600E detection, nine papillary thyroid cancer specimens were included in the study and their *BRAF* V600E mutation statuses were determined using Sanger sequencing, developed RT-qPCR and V600E-specific immunohistochemistry. Among them, six were found carrying the V600E mutation in Sanger sequencing, and all these sequencing-positive samples were also positive in RT-qPCR assay and V600E-specific immunohistochemistry (Fig. 2B, case 1). A low tumor content case being both RT-qPCR- and IHC-positive was found among the three V600E Sanger sequencing-negative samples (Fig. 2B, Case 2). With the V600E Sanger sequencing results of nine thyroid cancer samples, two V600E Sanger sequencing-positive and 39 V600E Sanger sequencing-negative lung cancer samples set as references for comparison with the RT-qPCR and V600E-specific immunohistochemistry results (Supplementary Table 1), the detection sensitivity, detection specificity, false-positive rate and false-negative rate of the developed RT-qPCR assay and V600E immunohistochemistry were both 100%, 97.6%, 11.1% and 0%, respectively.

**Table 1:** Clinicopathological features of lung cancer patients in the study.

Variable	Group	No. (%)
**Age**	Median	65
	Range	33–96
**Gender**	Male	164 (53.6)
	Female	142 (46.4)
**Smoking**^**#**^	Never or light	203 (66.3)
	Heavy	103 (33.7)
**Stage**	IA	36 (11.7)
	IB	74 (24.2)
	IIA	18 (5.9)
	IIB	18 (5.9)
	IIIA	44 (14.4)
	IIIB	15 (4.9)
	IV	101 (33.0)
**Histology**	Adeno	242 (79.1)
	Squamous	32 (10.4)
	Adenosquamous	7 (2.3)
	Large	6 (2.0)
	Sarcomatoid	5 (1.6)
	Others	14 (4.6)
**Total**		306 (100)

^#^Smoking status: never or light: 0–20 pack-year; heavy: > 20 pack-year.

## Discussion

Accurate molecular diagnosis is not only important for cancer treatment, but also for understanding in detail the clinicopathological features of cancers with specific type of mutation. To better understand the incidence and clinicopathological features of *BRAF* V600E/K mutation in lung cancer patients of southern Taiwan, a highly sensitive RT-qPCR method was established and 306 lung cancer samples were used for screening. The present results agree with earlier findings from other regions and countries, showing low incidence of the *BRAF* V600E mutation that tends to exist in adenocarcinoma. Although some studies concluded that the frequencies of *BRAF* mutations in various ethnic groups showed no significant difference^5^, the *BRAF* V600E mutation frequency seems to be significantly lower in East-Asian population (n = 89 of 11,465; 0.78%)^18–22^ than in the Caucasian population (n = 132 of 7114; 1.86%)^9,23–29^ (p-value < 0.00001). Although the frequency found in the current study (n = 2 of 306; 0.65%) has no significant difference to neither other East-Asian populations (p-value = 0.809) nor Caucasian population (*p*-value = 0.122), the mechanisms accounting for the ethnic difference are worth further investigation. Other features, including gender, smoking habit, and age of the identified positive cases in the current study do not fall within the preferential feature categories of *BRAF* V600E cases found in other studies. Although positive cases in this study are too few to make an explicit conclusion, the results did indicate that reporting clinicopathological features may not help much in patient enrichment for *BRAF* V600E screening, since the number of patients in the minority clinicopathological feature group is also substantial^7^, implying that detection at sequence level would be a better strategy for *BRAF* V600E screening.

Various strategies could be used for identifying of mutations at sequence level. The current practice before treatment involves screening a number of drugable mutations; hence, high-throughput techniques, such as next-generation sequencing, have been taken as an ultimate solution to the screening requirement. However, these high-throughput techniques are either technically more challenging or have not been widely applied and validated, greater caution is thus needed for establishing detailed consensus in experimental procedures and data processing^30–32^. The turnaround time and total cost of these high-throughput assays in each run could become satisfactory only when the sample volume and number of targets exceed certain threshold values. It would generally take several days for detection and data processing, and total costs could be even far beyond the expectation if the assays are performed by professional service providers. Moreover, the comprehensiveness of these newer techniques can even cause both false-positive and false-negative results due to nucleic acid deterioration caused by DNA crosslinking, abscission, and cytosine deamination during sample preservation, thus making result interpretation problematic^31,33^. Therefore, the currently used simple methodologies, such as RT-qPCR and immunohistochemistry, remain valuable for specific single-mutation screening, such as *BRAF* V600E mutation.

Compared with direct detection at sequence level for identifying mutations of interest, immunohistochemistry offers several advantages, such as being easy to perform, convenient and good single-cell level detection sensitivity, while reflecting faithfully the expression of functional mutant molecules, instead of the upstream coding mutant templates, as the most important. Although the BRAF V600E-specific antibody (clone VE1) has been widely used in *BRAF* V600E detection, false-positive and false-negative results are frequently observed in various types of cancers, including lung cancer^25,34–38^. The false-negative results are frequently attributed to non-optimized pre-analytical procedures or low tumor content in specimen, but the causes for false-positivity are still largely unknown. One study has reported that the epitope in the mutant BRAF V600E protein recognized by the VE1 antibody was found sharing high levels of sequence homology with several axonemal dynein heavy chain proteins (e.g., DNAH2, DNAH7, and DNAH12) in cilia^39^. However, the expressions of these proteins in lung cancers have not been thoroughly investigated, despite the important role played by cilia found in lung airway epithelial cells in WNT signaling^40,41^. Since the cilia of adjacent normal bronchial epithelial cells were also stained strongly by the V600E antibody (Supplementary Fig. S2) and ciliated adenocarcinomas of the lung has been reported before^42^, whether these axonemal dynein heavy chain proteins are overexpressed in subsets of lung cancer and mislead *BRAF* V600E-specific antibody detection requires more data to conclude. To avoid false signals being misinterpreted in V600E-specific immunohistochemistry, double confirmation using another efficient and cost-effective detection methods, such as the RT-qPCR assay developed herein, should be performed to help improve accuracy of *BRAF* V600E screening.

RT-qPCR coped with mutant specific primer is commonly used in molecular diagnosis. Despite tedious development, high sensitivity and specificity could be achieved after optimization. Moreover, it could even detect single-digit copies of target of interest surrounded by much more abundant wild-type counterparts as shown in the current and many other studies^43^. The detection sensitivity is comparable with that of other newer detection platforms, such as digital PCR and next generation sequencing^44^. Although the comprehensiveness of qPCR-based assay is far behind these newer high-throughput techniques, multi-target detection could be easily achieved through multiplexing or in panel fashion, and could still be able to screen multiple targets conveniently. Other advantages, including flexibility and scalability in detection, short turnaround time, straightforward readout, and affordable price, could make the newly developed RT-qPCR suitable for *BRAF* V600E/K screening. The newly developed highly sensitive RT-qPCR assay could also be directly implanted into digital PCR detection to better justify a small difference or obtain absolute quantitative data^45^. Of note is that the newly developed RT-qPCR assay would require additional assay to differentiate between V600E and V600K mutations, low-sensitivity assay, such as Sanger sequencing, should be adopted with caution downstream. Currently, authentic plasmid is widely adopted as the standard for quantitating the amount of mutant alleles in samples due to the convenience in preparation. Nevertheless, the much simpler sequence complexity in these plasmid-based standards could potentially overestimate the primer/probe specificity and the detection sensitivity even when supplemented with *E. coli* or yeast ribosomal RNA. Evaluation and validation of assay reagents and condition that mimics real clinical setting should be included before introducing any newly developed detection method into clinical practice.

In summary, a highly sensitive and *BRAF* V600E/K mutation-specific RT-qPCR was established for detection of *BRAF* V600E/K mutation and results found low incidence of the *BRAF* V600E mutation in the lung cancer population of southern Taiwan. Not all clinicopathological features of positive cases fall into the reported preferential clinicopathological feature group; hence, direct molecular diagnosis using the newly developed RT-qPCR and specific antibody is better for *BRAF* V600E/K mutation identification.

## Materials and methods

### Specimen collection

A total of 306 formalin-fixed paraffin-embedded lung cancer tissue samples collected between 2006 and 2018 with signed informed consent obtained from all participants or their legal guardians were either requested from the tissue bank and biobank of Chang Gung Memorial Hospital, Chiayi (193 samples) or collected prospectively for identification and validation of lung cancer biomarkers (113 samples). For validation of the newly developed RT-qPCR assay, nine specimens of papillary thyroid cancer, which potentially have higher frequency of *BRAF* V600E mutation, were also included in the study to validate the performance of the newly developed RT-qPCR assay. The study was conducted with approval from the institutional review board of Chang Gung Memorial Hospital (IRB approval No: 100-10405B, 201600915B0 and 201600631B0) and followed the guidelines of Human Subjects Research Act of Taiwan and *Declaration* of *Helsinki*. The clinicopathological features of these samples are summarized in Table 1.

### Genomic DNA purification

Genomic DNA of these lung cancer samples were respectively purified using AllPrep DNA/RNA Mini kit and QIAamp DNA FFPE Tissue Kit (Qiagen, Hilden, Germany) according to the manufacturer’s protocol.

### *BRAF* V600E/K specific real time-qPCR

The *BRAF* V600E/K RT-qPCR assay was modified from the protocol established by Lang et al.^17^. The relative locations and sequences of primers and probes used in the assay are illustrated in Fig. 1. Introducing a mismatched penultimate nucleotide (C to G) to the original mutant-reverse primer was found to be able to improve detection sensitivity and specificity^17^. Two separate qPCR reactions (wild-type and V600E mutant-specific) were performed to detect and measure the relative abundance of V600E allele in each sample. *CYP17* gene was chosen as the internal control. All qPCR reactions were performed using QuantiNova Probe PCR master mix (Qiagen, Hilden, Germany), with 900 nM *BRAF* mutant unspecific primer, 450 nM mutant-specific primer, 100 nM *BRAF* probe, 112.5 nM CYP17 internal control primer, 25 nM CYP17 internal control probe and 5 ng of sample DNA in a total volume of 20 μl on the Rotor-gene Q qPCR machine (Qiagen) under the following conditions: denaturation at 95 °C for 10 min, and 40 cycles of 95 °C for 15 s, 61 °C for 60 s. For each batch of assay, authentic standard containing two to 2000 copies of V600E or V600K mutant alleles prepared from blending of HEK293, and HT-29 or LM-MEL-42 genomic DNA. The normalized fluorescence intensity of 0.03 was employed to acquire the threshold cycle (Ct) data for analysis. All qPCR products were resolved in 4% agarose gel for result confirmation.

### *BRAF* V600 mutation determination by Sanger sequencing

To confirm and determine the V600 mutation type in *BRAF* V600E/K mutation-specific RT-qPCR positive samples, the DNA sequence around the *BRAF* V600 region was amplified from 5 ng genomic DNA using primer pair with following sequences 5′-TGTAAAACGACGGCCAGTCTGTTTTCCTTTACTTACTACACCTCAGAT-3′ and 5′-CAACTGTTCAAACTGATGGG-3, and GoTaq master mix (Promega, Madison, WI). An M13 forward primer adaptor sequence (labeled with underline) was incorporated into the forward primer to facilitate sequencing. The PCR product was then sent to sequencing using BigDye terminator (Applied Biosystems, Waltham, MA).

### V600E mutation-specific immunohistochemistry

V600E mutation-specific immunohistochemistry was performed on tissue microarray (TMA) or tumor tissue section. TMA was prepared for the 193 samples requested from tissue bank and biobank. For each case, 1.5-mm cores were sampled from two different tumor parts and one adjacent normal part for tissue microarray construction. Tumor tissue sections were made for prospectively enrolled patients carrying the *BRAF* V600E mutation. For detection of BRAF V600E mutant protein, the anti-V600E mutation-specific mouse monoclonal antibody (clone VE1, Ventana Medical Systems, Tucson, AZ) was used. Staining was carried out according to the manufacturer’s protocol on the BenchMark XT autostainer and signals were visualized using the Ventana OptiView DAB detection kit. Results obtained were interpreted by two pathologists blinded to the clinical information of these samples. Only signals of staining in the cytoplasm were interpreted as positive.

## Supplementary information

Supplementary Information.

## References

[1] Vogelstein B (2013). Cancer genome landscapes. Science.

[2] Yang F (2015). Protein domain-level landscape of cancer-type-specific somatic mutations. PLoS Comput. Biol..

[3] Fey D, Matallanas D, Rauch J, Rukhlenko OS, Kholodenko BN (2016). The complexities and versatility of the RAS-to-ERK signalling system in normal and cancer cells. Semin. Cell Dev. Biol..

[4] Khan AQ (2019). RAS-mediated oncogenic signaling pathways in human malignancies. Semin. Cancer Biol..

[5] Leonetti A (2018). BRAF in non-small cell lung cancer (NSCLC): Pickaxing another brick in the wall. Cancer Treat Rev.

[6] Wan PT (2004). Mechanism of activation of the RAF-ERK signaling pathway by oncogenic mutations of B-RAF. Cell.

[7] Cui, G. *et al.* A meta-analysis of the association between BRAF mutation and nonsmall cell lung cancer. *Medicine (Baltimore)* **96**, e6552, 10.1097/MD.0000000000006552 (2017). 10.1097/MD.0000000000006552 PMC5411210 28383426

[8] Litvak AM (2014). Clinical characteristics and course of 63 patients with BRAF mutant lung cancers. J. Thorac. Oncol..

[9] Marchetti A (2011). Clinical features and outcome of patients with non-small-cell lung cancer harboring BRAF mutations. J. Clin. Oncol..

[10] Dankner, M. Targeted therapy for colorectal cancers with non-V600 BRAF mutations: Perspectives for precision oncology. *JCO Precis. Oncol.* 1–12, 10.1200/po.18.00195 (2018). 10.1200/PO.18.00195 35135170

[11] Mazieres J (2020). Vemurafenib in non-small-cell lung cancer patients with BRAF(V600) and BRAF(nonV600) mutations. Ann. Oncol..

[12] Weart, T. C., Miller, K. D. & Simone, C. B., 2nd. Spotlight on dabrafenib/trametinib in the treatment of non-small-cell lung cancer: place in therapy. *Cancer Manag. Res.* **10**, 647–652, https://doi.org/10.2147/CMAR.S142269 (2018). 10.2147/CMAR.S142269 PMC5892608 29662327

[13] Planchard D (2016). Dabrafenib plus trametinib in patients with previously treated BRAFV600E-mutant metastatic non-small cell lung cancer: An open-label, multicentre phase 2 trial. Lancet Oncol..

[14] Planchard D (2017). Dabrafenib plus trametinib in patients with previously untreated BRAFV600E-mutant metastatic non-small-cell lung cancer: An open-label, phase 2 trial. Lancet Oncol..

[15] Noeparast A (2017). Non-V600 BRAF mutations recurrently found in lung cancer predict sensitivity to the combination of Trametinib and Dabrafenib. Oncotarget.

[16] Subbiah, V. *et al.* Efficacy of Vemurafenib in patients with non-small-cell lung cancer with BRAF V600 mutation: An open-label, single-arm cohort of the histology-independent VE-BASKET study. *JCO Precis. Oncol.* 1–9, 10.1200/po.18.00266 (2019). 10.1200/PO.18.00266 PMC7446432 32914022

[17] Lang AH (2011). Optimized allele-specific real-time PCR assays for the detection of common mutations in KRAS and BRAF. J. Mol. Diagn..

[18] Kinno T (2014). Clinicopathological features of nonsmall cell lung carcinomas with BRAF mutations. Ann. Oncol..

[19] Kobayashi M (2011). Clinical significance of BRAF gene mutations in patients with non-small cell lung cancer. Anticancer Res..

[20] Lee SY (2010). Somatic mutations in epidermal growth factor receptor signaling pathway genes in non-small cell lung cancers. J. Thorac. Oncol..

[21] Lin Q (2019). The association between BRAF mutation class and clinical features in BRAF-mutant Chinese non-small cell lung cancer patients. J. Transl. Med..

[22] Sasaki H (2012). Braf and erbB2 mutations correlate with smoking status in lung cancer patients. Exp. Ther. Med..

[23] Brustugun OT (2014). BRAF-mutations in non-small cell lung cancer. Lung Cancer.

[24] Cardarella S (2013). Clinical, pathologic, and biologic features associated with BRAF mutations in non-small cell lung cancer. Clin. Cancer Res..

[25] Ilie M (2013). Diagnostic value of immunohistochemistry for the detection of the BRAFV600E mutation in primary lung adenocarcinoma Caucasian patients. Ann. Oncol..

[26] Luk PP (2015). BRAF mutations in non-small cell lung cancer. Transl. Lung Cancer Res..

[27] Paik PK (2011). Clinical characteristics of patients with lung adenocarcinomas harboring BRAF mutations. J. Clin. Oncol..

[28] Schmid K (2009). EGFR/KRAS/BRAF mutations in primary lung adenocarcinomas and corresponding locoregional lymph node metastases. Clin. Cancer Res..

[29] Tissot C (2016). Clinical characteristics and outcome of patients with lung cancer harboring BRAF mutations. Lung Cancer.

[30] Bacher U (2018). Challenges in the introduction of next-generation sequencing (NGS) for diagnostics of myeloid malignancies into clinical routine use. Blood Cancer J..

[31] Do H, Dobrovic A (2015). Sequence artifacts in DNA from formalin-fixed tissues: causes and strategies for minimization. Clin. Chem..

[32] Yip S (2019). A Canadian guideline on the use of next-generation sequencing in oncology. Curr. Oncol..

[33] Haile S (2019). Sources of erroneous sequences and artifact chimeric reads in next generation sequencing of genomic DNA from formalin-fixed paraffin-embedded samples. Nucleic Acids Res..

[34] Behling F (2016). Frequency of BRAF V600E mutations in 969 central nervous system neoplasms. Diagn. Pathol..

[35] Dvorak K (2014). Immunohistochemistry with the anti-BRAF V600E (VE1) antibody: Impact of pre-analytical conditions and concordance with DNA sequencing in colorectal and papillary thyroid carcinoma. Pathology.

[36] Gow, C. H., Hsieh, M. S., Lin, Y. T., Liu, Y. N. & Shih, J. Y. Validation of immunohistochemistry for the detection of BRAF V600E-mutated lung adenocarcinomas. *Cancers (Basel)* **11**, 10.3390/cancers11060866 (2019). 10.3390/cancers11060866 PMC6627826 31234388

[37] Lee SR (2015). VE1 antibody is not highly specific for the BRAF V600E mutation in thyroid cytology categories with the exception of malignant cases. Am. J. Clin. Pathol..

[38] Schirosi L (2016). Is immunohistochemistry of BRAF V600E useful as a screening tool and during progression disease of melanoma patients?. BMC Cancer.

[39] Jones RT (2015). Cross-reactivity of the BRAF VE1 antibody with epitopes in axonemal dyneins leads to staining of cilia. Mod. Pathol..

[40] Higgins M, Obaidi I, McMorrow T (2019). Primary cilia and their role in cancer. Oncol. Lett..

[41] Jamal, M. H. & Nauli, S. Primary cilia in cancer cells. *FASEB J.* **33**, 815.817–815.817, 10.1096/fasebj.2019.33.1_supplement.815.7 (2019).

[42] Park WY (2012). Ciliated adenocarcinomas of the lung: A tumor of non-terminal respiratory unit origin. Mod. Pathol..

[43] Matsuda K (2017). PCR-based detection methods for single-nucleotide polymorphism or mutation: Real-time PCR and its substantial contribution toward technological refinement. Adv. Clin. Chem..

[44] Dong L, Wang S, Fu B, Wang J (2018). Evaluation of droplet digital PCR and next generation sequencing for characterizing DNA reference material for KRAS mutation detection. Sci. Rep..

[45] Huggett JF, Cowen S, Foy CA (2015). Considerations for digital PCR as an accurate molecular diagnostic tool. Clin. Chem..

