# Author Correction: A highly sensitive and specific real-time quantitative PCR for *BRAF* V600E/K mutation screening

**DOI:** 10.1038/s41598-021-92318-5

**Published:** 2021-06-15

**Authors:** Jrhau Lung, Ming‑Szu Hung, Yu‑Ching Lin, Yuan Yuan Jiang, Yu‑Hung Fang, Ming‑Shian Lu, Ching‑Chuan Hsieh, Chia‑Siu Wang, Feng‑Che Kuan, Chang‑Hsien Lu, Ping‑Tsung Chen, Chieh‑Mo Lin, Yen‑Li Chou, Chin‑Kuo Lin, Tsung‑Ming Yang, Fen Fen Chen, Paul Yann Lin, Meng‑Jer Hsieh, Ying Huang Tsai

**Affiliations:** 1 Department of Medical Research and Development, Chang Gung Memorial Hospital, Chiayi Branch, Chiayi, Taiwan; 2 Department of Pulmonary and Critical Care Medicine, Chang Gung Memorial Hospital, Chiayi Branch, Chiayi, Taiwan; 3 Department of Medicine, College of Medicine, Chang Gung University, Taoyuan, Taiwan; 4 Department of Respiratory Care, Chang Gung University of Science and Technology, Chiayi Campus, Chiayi, Taiwan; 5 Division of Thoracic and Cardiovascular Surgery, Department of Surgery, Chiayi Branch, Chang Gung Memorial Hospital, Chiayi, Taiwan; 6 Department of General Surgery, Chang Gung Memorial Hospital, Chiayi Branch, Chiayi, Taiwan; 7 Department of Hematology and Oncology, Chang Gung Memorial Hospital, Chiayi Branch, Chiayi, Taiwan; 8 Department of Pathology, Chang Gung Memorial Hospital, Chiayi Branch, Chiayi, Taiwan; 9 Department of Anatomic Pathology, Dalin Tzu Chi Hospital, Buddhist Tzu Chi Medical Foundation, Chiayi, Taiwan; 10 Department of Respiratory Care, College of Medicine, Chang Gung University, Taoyuan, Taiwan; 11 Department of Pulmonary and Critical Care Medicine, Chang Gung Memorial Hospital, Linkou Branch, Linkou, Taiwan

Correction to: *Scientific Reports* 10.1038/s41598-020-72809-7, published online 09 October 2020

The Acknowledgements section of this Article was incomplete.

“We would like to thank Precious Instrumentation Core Laboratory, Department of Medical Research and Development, Chang Gung Memorial Hospital, Chiayi for providing the tissue microarray constructed using the AutoTiss 1000 tissue microarrayer (EverBio, New Taipei City, Taiwan, ROC).”

now reads:

“We would like to thank Precious Instrumentation Core Laboratory, Department of Medical Research and Development, Chang Gung Memorial Hospital, Chiayi for providing the tissue microarray constructed using the AutoTiss 1000 tissue microarrayer (EverBio, New Taipei City, Taiwan, ROC). The study was supported by the Grant No: CORPG6F0021-3 and CMRPG6F0471-3 from the Chang Gung Memorial Hospital, Chiayi, Taiwan.”

The original Article has been corrected.

