# Supplementary material for: A highly sensitive and specific real-time quantitative PCR for BRAF V600E/K mutation screening
Source: Sci Rep. 2020 Oct 9;10:16943. doi: 10.1038/s41598-020-72809-7 (PMC7547094; doi:10.1038/s41598-020-72809-7)
Supplement: Supplementary file 1 — Supplementary Information. [file 41598_2020_72809_MOESM1_ESM.pdf]

A highly sensitive and specific real-time quantitative PCR for *BRAF* V600E/K mutation screening

Jrhau Lung[1], Ming-Szu Hung[2,3,4], Yu-Ching Lin[2,3,4], Yuan Yuan Jiang[2], Yu-Hung Fang[2], Ming-Shian Lu[5], Ching-Chuan Hsieh[6], Chia-Siu Wang[6], Feng-Che Kuan[7], Chang-Hsien Lu[7], Ping-Tsung Chen[7], Chieh-Mo Lin[2], Yen-Li Chou[2], Chin-Kuo Lin[2], Tsung-Ming Yang[2], Fen Fen Chen[8], Paul Yann Lin[9], Meng-Jer Hsieh[2,10],Ying Huang Tsai[2,10,11*]

[1]Department of Medical Research and Development, Chang Gung Memorial Hospital, Chiayi Branch, Taiwan

[2]Department of Pulmonary and Critical Care Medicine, Chang Gung Memorial Hospital, Chiayi Branch, Taiwan

[3]Department of Medicine, College of Medicine, Chang Gung University, Taoyuan, Taiwan

[4]Department of Respiratory Care, Chang Gung University of Science and Technology, Chiayi Campus, Chiayi, Taiwan.

[5]Department of Surgery, Division of Thoracic and Cardiovascular Surgery, Chang Gung Memorial Hospital, Chiayi Branch, Taiwan

[6]Department of General Surgery, Chang Gung Memorial Hospital, Chiayi Branch, Taiwan

[7]Department of Hematology and Oncology, Chang Gung Memorial Hospital, Chiayi Branch, Taiwan

[8]Departmeny of Pathology, Chang Gung Memorial Hospital, Chiayi Branch, Taiwan.

[9]Departmeny of Anatomic Pathology, Dalin Tzu Chi Hospital, Buddhist Tzu Chi Medical Foundation, Chiayi, Taiwan.

[10]Department of Respiratory Care, College of Medicine, Chang Gung University, Taoyuan, Taiwan

[11]Department of Pulmonary and Critical Care Medicine, Chang Gung Memorial Hospital, Linkou Branch, Taiwan

Correspondence: Ying Huang Tsai (email:chestmed@cgmh.org.tw)

Supplementary Figure S1

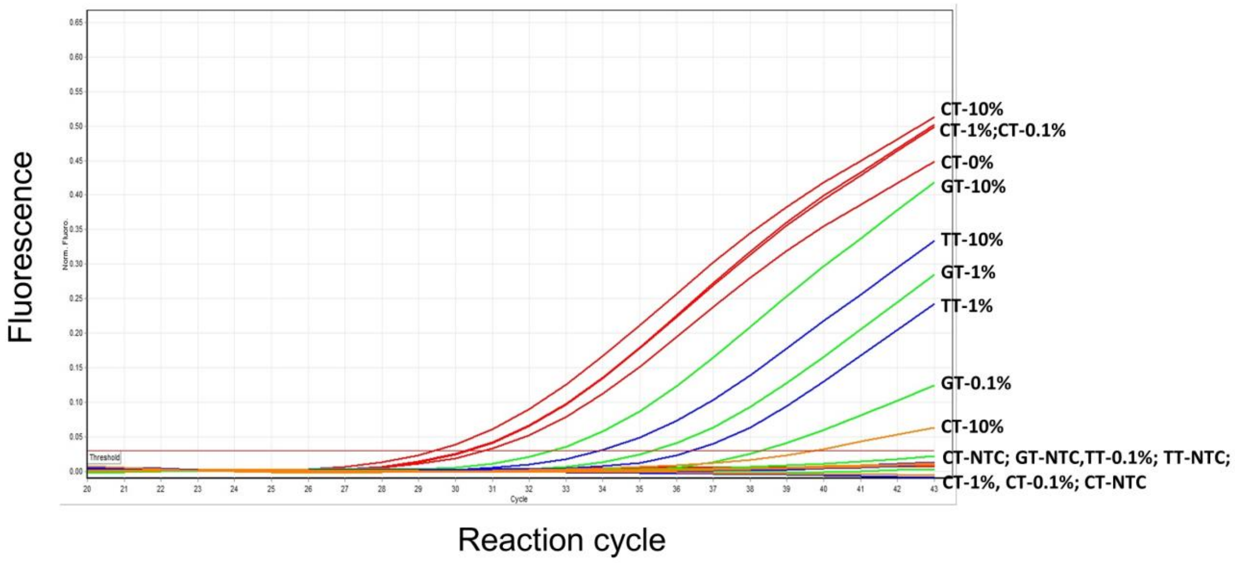

Influence of introducing various mismatched penultimate nucleotides to the *BRAF* V600E mutant-reverse primers on V600E detection performance. These primers were tested using standards containing 0-10% BRAF V600E mutant allele prepared from HEK293 and HT-29 genomic DNA. Colors of qPCR curves in the amplification plot were labeled according to the last two nucleotides in *BRAF* V600E mutant-reverse primers. The original mutant-reverse primer ended with CT was labeled in red. Newly designed primers ended with GT, TT and CT were labeled with green, blue and orange, respectively. The end of each qPCR amplification curve was labeled with last two nucleotides of each mutant-reverse primer and the concentration of *BRAF* V600E mutant alleles in standard used in each assay to facilitate data interpretation.

Supplementary Figure S2

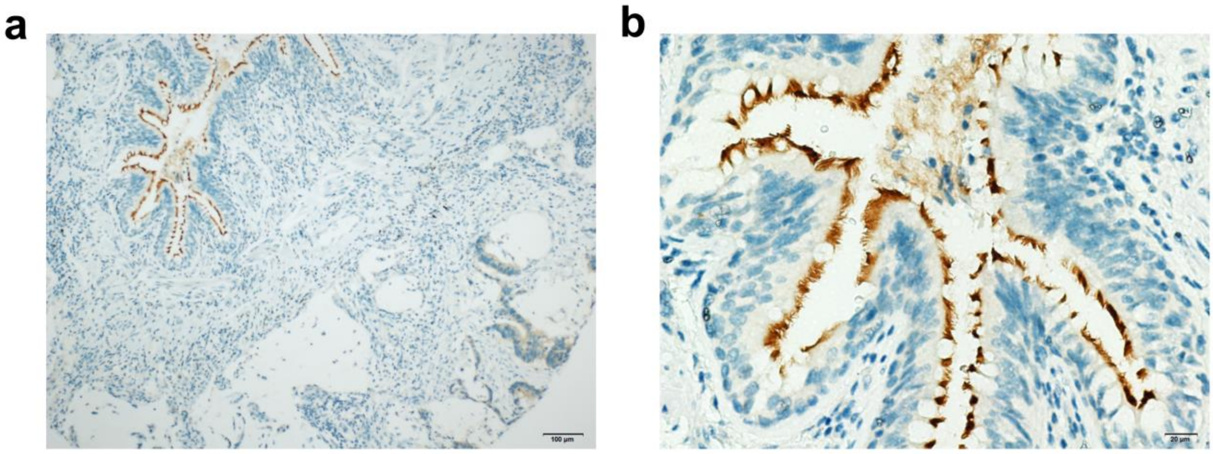

Supplementary Figure S2 Non-specific staining of V600E specific antibody on the cilia of bronchial epithelial cells. (A) The cilia of bronchial epithelial cells and tumor cells of the *BRAF* V600E mutation positive case 1 were both stained by the BRAF V600E specific antibody. (B) A magnified image of the stained bronchial epithelial cells.

Supplementary Table 1. The *BRAF* V600E mutation statuses in selected lung and thyroid cancer specimens obtained by Sanger sequencing, RT-qPCR and V600E immunohistochemistry for determining detection sensitivity and specificity.

Cancer type	Assay result	Sanger sequencing	RT-qPCR	V600E IHC
Lung cancer	+	2	2	2
	-	39	39	39
Thyroid cancer	+	6	7	7
	-	3	2	2
